# The impact of wait time on patient outcomes in knee and hip replacement surgery: a scoping review protocol

**DOI:** 10.1186/s13643-022-01909-4

**Published:** 2022-03-04

**Authors:** E. Dawson, M. E. Neufeld, E. Schemitsch, A. John-Baptiste

**Affiliations:** 1 Department of Epidemiology & Biostatistics, Schulich School of Medicine and Dentistry, Western University, London, Ontario N6A 5B8 Canada; 2 Department of Orthopaedics, Faculty of Medicine, University of British Columbia, Vancouver, British Columbia Canada; 3 Department of Orthopaedics, Complex Joint Reconstruction Clinic, Gordon & Leslie Diamond Health Care Centre, University of British Columbia, 3rd Floor, 2775 Laurel Street, Vancouver, British Columbia V5Z 1M9 Canada; 4 Department of Surgery, Schulich School of Medicine & Dentistry, London, Ontario Canada; 5 London Health Sciences Centre, 800 Commissioners Road East, PO Box 5010, Stn B, London, Ontario N6A 5W9 Canada; 6 Department of Anesthesia & Perioperative Medicine, Schulich School of Medicine and Dentistry, Western University, London, Ontario Canada; 7 Schulich Interfaculty Program in Public Health, Western University, London, Ontario Canada

**Keywords:** Wait times, Total hip replacement, Total knee replacement, Patient reported outcomes, Scoping review, Perioperative period, Quality of life, Postoperative outcomes

## Abstract

**Background:**

Total hip and total knee replacement surgery are in high demand, leading to long wait times for many patients. While on the waiting list, patients may experience worsening pain, reduced mobility, and deteriorating health. Given that long wait times are common for lower joint replacement surgery, it is important to understand how patient health changes during the wait period and whether this impacts patient outcomes after surgery. The aim of this scoping review will be to identify and describe the evidence regarding the impact of wait time on patient outcomes for patients who undergo total knee and total hip replacement surgery.

**Methods:**

This scoping review was designed with guidance from the Joanna Briggs Institute Manual for Evidence Synthesis, and results will be reported following the Preferred Reporting Items for Systematic Reviews and Meta-Analyses extension for scoping reviews. EMBASE, Medline, PubMed, Scopus, CINAHL, and Cochrane electronic databases will be searched for English language articles published after 1999. Studies of adult patients with osteoarthritis undergoing primary knee or hip replacement surgery, which measure patient outcomes over the wait period for surgery, will be included. Two independent reviewers will screen titles and abstracts followed by full article review. Data will be extracted by two reviewers using a standardized form. Outcomes assessed during the wait period will be identified and described in tables. Factors associated with changes in health status during the wait period will be qualitatively described.

**Discussion:**

This review will map the evidence regarding wait times for lower extremity joint replacement surgery. Better understanding of how the impact of wait times on patient health status is measured over the perioperative period will inform future research on wait times.

**Scoping review registration:**

Registered with Open Science Framework, Feb 14, 2021 DOI: 10.17605/OSF.IO/MV4FS

## Background

Total hip replacements and total knee replacements are among the most commonly performed surgeries, and the number of procedures performed annually is increasing for many countries [1, 2]. High demand for these surgeries can lead to significant wait times and corresponding wait lists; as such, elective knee and hip replacements were identified as priority procedures for reducing wait times in 2004 [3]. Despite this, wait times for elective joint replacement surgeries remain an issue. In 2018 and 2019, only 72% of patients in Canada received their knee or hip replacement within the benchmark time of 26 weeks, and some patients are still waiting longer than 1 year to undergo surgery [4]. The current COVID-19 pandemic is further contributing to long wait times by limiting the capacity of hospitals to perform elective surgeries, and this may have lasting effects on wait times for joint replacement surgery in the coming years [5]. The primary indication for total joint replacement of the knee or hip is osteoarthritis that cannot be managed with nonsurgical interventions [1, 2]. Osteoarthritis causes pain and stiffness in the joint leading to reduced function and poor quality of life for patients [6]. Patients experience pain and reduced mobility, while awaiting surgery and after prolonged wait periods, these symptoms may worsen.

In a typical referral pathway for patients who undergo hip and knee replacement surgery, patients initially see their primary health provider and are referred to an outpatient orthopedic clinic for physical assessment and diagnostic imaging tests [7]. Once a decision to undergo surgery is made, the patient is placed on a waiting list. Once on a waitlist, patients have little to no contact with healthcare providers until the time of surgery, meaning changes in patient health during this time may go undetected [8]. Understanding the trajectories of patient health status while on the wait list could help clinical decision-makers characterize the risk of deterioration of patient outcomes while awaiting surgery and the risk of poor outcomes after joint replacement surgery [9].

For patients undergoing joint replacement, there are a number of possible outcome measures that can be used to assess changes in patients’ condition over time and the impact that the surgery has on symptoms, function, and overall health of patients [10]. Revision surgery, required when patients face serious postsurgical complications such as pain, infection, or implant loosening and wear [1], is an important clinical outcome measure. However, revision rates are generally very low and do not necessarily reflect patient satisfaction or experience with symptoms [1, 11]. Therefore, in recent years, the focus of researchers and clinicians has shifted toward assessing outcomes from the patient perspective.

Patient-reported outcome measures (PROMs) are instruments completed by patients to provide information on their physical, mental, and social health and quality of life [11]. PROMs can be either generic or disease specific; generic PROMs can be applied to the general population and are usually concerned with health-related quality of life [12]. Disease-specific PROMs are applied to populations with a particular disease or symptoms. For osteoarthritis, specific PROMs such as the Western Ontario and McMaster Universities Arthritis Index (WOMAC) or the Oxford Knee Score have been used to assess the severity of symptoms [11, 12].

Patient outcomes may also be assessed directly by orthopedic surgeons or other clinicians. The Harris Hip Score, for example, is a questionnaire designed to be completed by clinicians to assess a patient’s pain, function, and mobility [13]. Clinicians may also conduct radiological assessments or performance tests such as the timed get-up-and-go or the 20-m walk test [10, 13]. Performance-based assessments can give objective measures of function and can be used to assess changes in function over time [10].

A search for existing scoping or systematic reviews on wait times for joint replacement surgery identified two previously published reviews. A 2009 systematic review from Hoogeboom et al. investigated the association between wait time for total joint replacements and patient pain and function [14]. The authors concluded that with wait times less than 180 days, there was no deterioration in functional status or increase in pain, and that the evidence was uncertain regarding deterioration for wait times longer than 180 days [14]. A more recent scoping review, published by Morris et al. in 2018, described the impact of waiting for knee and hip replacement surgery on quality of life [8]. The review found that numerous tools were used to assess quality of life at variable timepoints across the wait period and after surgery. The evidence of the impact of waiting was inconsistent, and the authors concluded that changes in quality of life during the wait period may be impacted by choice of outcome measure and patient characteristics and called for more research into measures used to assess the impact of waiting for joint surgery [8].

Given the inconclusive findings of these reviews and the fact that it has been 5 years since a search was conducted on this topic [8, 14], an updated review of the evidence around wait times for joint replacement surgery is needed. Furthermore, the previously conducted reviews were each focused on the impact of wait times on specific patient outcomes; however, given the wide range of outcome assessments possible in joint replacement surgery, the impact of wait times may be studied in a variety of ways [10]. Therefore, we propose a more expansive search, not limited by outcome, that may identify literature not captured in previous reviews or that has been published in recent years. The delays to elective surgery, precipitated by the Covid-19 pandemic, may have generated new interest in wait time research since 2020.

A scoping review design is proposed because we expect heterogeneity across studies in their conceptualization of the wait period and in how patient health changes are measured while waiting. Therefore, we will focus on describing these concepts in order to better understand the evidence for the impact of wait times in joint replacement patients. We will explore how research on wait time is conducted, identify gaps in the literature, and map the evidence across a wide selection of patient outcomes. The aim of this scoping review is to describe how changes in patient health while waiting for surgery are reported and to describe how the impact of wait time on preoperative and postoperative outcomes is assessed, for patients who undergo total knee and total hip replacement.

### Objectives

1. To identify the outcomes that are used to evaluate the impact of wait time on patients scheduled to undergo lower extremity joint replacement surgery.
2. To describe how outcome measures are employed to assess changes in patient health status while on the wait list, including when they are administered, specific metrics reported for each tool (i.e., domain measures vs summary scores), and whether any floor or ceiling effects were observed.
3. To report the trajectory of patient outcomes and performance measure scores over the wait period, through surgery, and into the postoperative period.
4. To identify studies that reported the influence of patient characteristics on the association between wait time and patient outcomes during the wait period and after surgery.

## Methods

This protocol has been registered with Open Science Framework DOI:10.17605/OSF.IO/MV4FS. The protocol for this review was developed with guidance from the Joanna Briggs Institute Manual for Evidence Synthesis [15]. The results of the review will be reported according to the Preferred Reporting Items for Systematic reviews and Meta-Analyses extension for scoping reviews (PRISMA-ScR) checklist [16].

### Eligibility criteria

#### Population

Studies of adult patients (aged 18 years or older) who are scheduled to undergo a primary total knee or total hip replacement, where the main indication for surgery is osteoarthritis of the knee or hip, will be eligible for inclusion. Studies will qualify for inclusion if 80% or more of the study population is diagnosed with osteoarthritis [14]. Studies that investigate patients undergoing revision joint replacement surgeries will be excluded.

#### Exposure

The exposure will be time waiting for surgery. Studies will be included if they provide a measure of the entire wait time with a defined start (e.g., referral by doctor or added to a wait list) and a defined end to the wait period (e.g., surgery or pre-admission clinic visit). Cross-sectional studies that only assess patient outcomes at one time point while on the wait list will be excluded. Studies that only report on a short period of the overall wait time will be excluded.

#### Outcome

We will include studies that assess outcomes at a minimum of two time points in the wait period before surgery and studies that also report on outcomes after surgery. Studies that assess any outcomes will be included. This may include, but is not limited to, patient-reported outcome measures, clinician-reported outcomes, performance-based measures, clinical assessments, measures of patient satisfaction, joint revisions, and other postsurgical adverse events.

#### Study characteristics

Studies published from the year 2000 until present will be included. The year 2000 was chosen as there have been no major changes to total joint replacement procedures in this time [17], and wait times have been an issue of interest over the past two decades [9]. Studies without a full text available in English will be excluded. If relevant abstracts are found, we will search for a subsequent publication to include. Abstracts without a full publication will be excluded. Both prospective and retrospective observational studies and randomized trial study designs will be included. Case reports, case-series, review articles, editorials, non-peer-reviewed articles and any other publications that do not include primary research will be excluded.

### Information sources

EMBASE, Medline, PubMed, Scopus, CINAHL, and Cochrane electronic databases will be searched. We will also search for theses and dissertations using the ProQuest and Networked Digital Library of Theses and Dissertations databases, and clinicaltrials.gov will be searched for relevant registered trials.

### Search strategy

Search terms for “total hip replacement,” “total knee replacement,” and “wait time” will be used to search each database. A full search strategy for the EMBASE database can be found in Table 1. Search terms related to the outcome of interest will not be included so that the search is inclusive of all possible outcomes, which may be referred to with a wide array of terminology in the existing literature. In addition to the database search, we will conduct forward and backward citation tracing for any relevant review articles identified in the database search to find additional articles not captured in the database search.

**Table 1:** Sample search strategy for the EMBASE database

Joint replacement	Wait time
Total hip replacement mp. Hip replacement mp. Hip arthroplasty mp. Total hip prosthesis mp. Total knee replacement mp. Knee replacement mp. Knee arthroplasty mp. Total knee prosthesis mp. Knee prosthesis mp. Hip prosthesis mp. Total joint replacement mp. Total arthroplasty Replacement arthroplasty Joint replacement mp. Joint replacement surgery mp. Knee osteoarthritis Hip osteoarthritis Osteoarthritis mp.	Wait* Await* Delay mp.

*indicates truncated search term

### Selection of sources

Study records will be managed using Covidence software for all levels of screening. Data extraction will be done in Microsoft Excel using an extraction form developed by the reviewers. Search results from each database will be uploaded to Covidence, and duplicate papers will be removed. Two independent reviewers will conduct screening by title and abstract and eliminate papers that do not meet inclusion criteria. Two independent reviewers will conduct full text screening of the remaining articles. Any disagreements between the reviewers will be resolved by a third reviewer.

### Data charting

A data extraction form will be created in Microsoft Excel. Data to be extracted will include study characteristics, such as year, setting, sample size, and design; patient characteristics such as age, sex, BMI, and comorbidities; details of the surgical procedure; and details of all patient-reported outcomes measured during the wait period and after surgery. Outcome-related data will include the measurement tools used and type of outcomes measured, the mean outcome scores at any time point, changes in outcomes scores, and any floor and ceiling effects reported for the measurement tools used. Data extraction will be performed by one reviewer and confirmed by a second reviewer. Any disagreements between the reviewers will be resolved by a third reviewer.

### Data items

A full list of data items that will be extracted are listed in the proposed data extraction form in Table 2.

**Table 2:** A list of data items that will be extracted from studies included in this review

Study characteristics	Author name, year Location Study design Data collection years Objective
Participants characteristics	Sample source Eligibility criteria Number recruited Sample size Reason lost to follow-up Mean and standard deviation (SD) or median and interquartile range (IQR) Age Sex Percent with osteoarthritis Comorbidities BMI Osteoarthritis severity Baseline functional assessments/ADLs/other Socioeconomic status
Wait time reported	Start of wait time measurement End of wait time measurement Mean and SD or median and IQR wait time Range of wait time Categories of wait time (i.e., < 6 months, > 6 months)
Outcomes measured	Outcomes intended to be measured Assessment method Modifications to the assessment method Domain measured (i.e., pain, function, quality of life) Possible score range
Outcome scores	Mean and SD or median and IQR baseline score Mean and SD or median and IQR follow-up score at all time points measured Change in mean/median score from baseline to final follow-up Floor effects identified Ceiling effects identified Proportion of subjects with negative change (worsening of outcome) Proportion of subjects with positive change (improvement of outcome) Proportion of subjects with no clinically important change
Factors associated with outcome change	Length of wait time Patient characteristics Health system factors

### Synthesis of results

We will descriptively synthesize the results of included studies; as this is a scoping review, no statistical analyses will be performed. Characteristics of the included studies will be summarized in tabular format including the date and location of the study, study design, and characteristics of the study population. We will describe the different ways that studies report on the waiting period and discuss any inconsistencies across the field that may influence the synthesis of study findings. We will describe how studies conceptualize the impact of wait time on patient health status before surgery and on health status after surgery. Outcomes will be categorized by type and by domain being assessed (i.e., pain, satisfaction, mobility). For studies that use specific measurement tools to assess outcomes, differences in how these tools were used across studies will be described. Changes in outcome scores and performance measures during the wait period will be reported. For studies that compare preoperative and postoperative outcomes, we will also report this change. We will summarize data on the trajectory of measures for studies that report outcome scores for two or more time periods. For example, the studies may demonstrate a stable trajectory, decreasing scores, and increasing scores suggest ceiling effects (measures with consistently high scores despite evidence of deterioration) or floor effects (measures with consistently low scores despite evidence of improvements). For studies that identify a wait time threshold beyond which measures deteriorate, we will report the threshold. Evidence of associations between patient characteristics and change in outcome scores will be qualitatively described.

## Discussion

This scoping review will map the evidence of the impact of waiting time on patients who undergo lower joint replacement surgery. Specifically, we will identify and describe the range of outcome measures that are used to assess patient health status during the wait period. This scoping review will not place limits on the outcomes to be included which will give a broad overview of the wait time literature. While a similar scoping review of wait time literature was recently published in 2018, the purpose of this review was to assess the impact of waiting for any orthopedic treatment on patient quality of life [8]. Furthermore, the results were focused on the timing of quality-of-life assessment and categorizing outcomes into positive, negative, or no significant change during the wait period. This proposed scoping review will build on this earlier work by conducting a more inclusive, updated search of the topic and by reporting in more detail on the outcome measures used to assess patient health status during the wait period. We will also identify patient or health system characteristics that are associated with deterioration while waiting for surgery, which was reported by the 2009 systematic review but not assessed in the 2018 scoping review [14]. Better understanding of the types of outcome measures used and the way they are applied during the perioperative period can help inform future research on the impact of wait times. This scoping exercise will identify the most relevant outcomes for which data can be synthesized, which will inform the feasibility of conducting a future systematic review and meta-analysis on the wait time literature.

We anticipate some potential challenges in reviewing the wait times literature for lower joint replacement surgery. The range of outcome measures (clinical outcomes, PROMs, performance measures) overlaid with a range of assessment time points could make it difficult to synthesize evidence or identify consistent trends. Indeed, concerns about identifying consistent measures and time points are in our view, the main reason for proposing a scoping review to profile the evidence landscape and gain further insight into the feasibility and appropriateness of conducting a systematic review and meta-analysis of the evidence.

Even if outcome measures are consistently reported at the same time points, study differences may contribute to heterogeneity that can complicate interpretation of study results. Heterogeneity may come from differences in patient characteristics, such as differences in age, comorbidities, socioeconomic status, and baseline measures. Heterogeneous patient populations may result in different wait time trajectories. To assess the potential for heterogeneity, we will record patient characteristics, in order to detect differences amongst study populations that would suggest the populations aren’t comparable. Heterogeneity resulting from health system factors, such as the availability of primary health care, use of supplemental interventions, and provision of social supports, may also make it challenging to interpret the scoping review findings. We will collect information from each study on health and social service utilization of the study population, to catalogue potential sources of heterogeneity in patient outcomes due to health system differences.

The findings of this review will catalogue wait time related outcome measures for patients undergoing lower joint replacement, summarize information on the trajectory of change in outcome measures, and identify wait time thresholds where patients are at risk for negative outcomes. The resulting information may guide clinical decision-making on the types of patient outcome assessments that should be used during the wait period. The resulting information can also promote greater consistency in how patient health status is measured during the wait period, which can ultimately improve the ability of the research community to identify important changes in patient health status during the wait period and possibly lead to policy changes that can improve outcomes of lower joint replacement surgery.

## Data Availability

Data sharing is not applicable to this protocol.
